# Antioxidant Properties of Whole Body Periodic Acceleration (pGz)

**DOI:** 10.1371/journal.pone.0131392

**Published:** 2015-07-02

**Authors:** Arkady Uryash, Jorge Bassuk, Paul Kurlansky, Francisco Altamirano, Jose R. Lopez, Jose A. Adams

**Affiliations:** 1 Division of Neonatology, Mount Sinai Medical Center, Miami Beach, Florida, United States of America; 2 Department of Molecular Biosciences, University of California Davis, Davis, California, United States of America; 3 Department of Surgery, Columbia University, New York, New York, United States of America; Universidad Pablo de Olavide, Centro Andaluz de Biología del Desarrollo-CSIC, SPAIN

## Abstract

The recognition that oxidative stress is a major component of several chronic diseases has engendered numerous trials of antioxidant therapies with minimal or no direct benefits. Nanomolar quantities of nitric oxide released into the circulation by pharmacologic stimulation of eNOS have antioxidant properties but physiologic stimulation as through increased pulsatile shear stress of the endothelium has not been assessed. The present study utilized a non-invasive technology, periodic acceleration (pGz) that increases pulsatile shear stress such that upregulation of cardiac eNOS occurs, We assessed its efficacy in normal mice and mouse models with high levels of oxidative stress, e.g. Diabetes type 1 and *mdx* (Duchene Muscular Dystrophy). pGz increased protein expression and upregulated eNOS in hearts. Application of pGz was associated with significantly increased expression of endogenous antioxidants (Glutathioneperoxidase-1(GPX-1), Catalase (CAT), Superoxide, Superoxide Dismutase 1(SOD1). This led to an increase of total cardiac antioxidant capacity along with an increase in the antioxidant response element transcription factor Nrf2 translocation to the nucleus. pGz decreased reactive oxygen species in both mice models of oxidative stress. Thus, pGz is a novel non-pharmacologic method to harness endogenous antioxidant capacity.

## Introduction

Redox signaling, defined as the reversible oxidation/reduction modification of cellular signaling pathways by reactive species is an important process in many physiological and pathophysiological states [1]. In the heart and vasculature, redox signaling is involved in excitation-contraction coupling (ECC), cell differentiation, stress response pathways, e.g., adaptation to hypoxia/ischemia and pathological processes, adverse cardiac remodeling, fibrosis, and atherosclerosis [2–4]. Reactive oxygen species (ROS) include free oxygen radicals, oxygen ions and peroxides. ROS at low to moderate concentrations regulate vascular tone, oxygen sensing, cell growth and proliferation, apoptosis, and inflammatory responses. Excessive or sustained ROS production, when exceeding the available antioxidant defense systems, produces oxidative stress that damages cell structure and disrupts function through lipid peroxidation of cell membranes, degrades nucleic acids [5]. Oxidative damage to cells and tissues is involved in the aging process and in chronic diseases including atherosclerosis, heart failure and cancer among others. Endogenously occurring protective antioxidants Glutathioneperoxidase-1 (GPX1), Superoxide Dismutase-1 (SOD-1, Cu-Zn SOD), and Catalase (CAT) maintain the balance of oxidizing chemicals, thereby playing a vital role in reduction of oxidative stress [1]. Epidemiological data suggest that diets rich in antioxidants have a protective effect on the development of cardiovascular disease. However, clinical trials and large meta-analysis have failed to show evidence for support of antioxidant supplements for prevention of cardiovascular disease and suggest potential deleterious effects [6–8]. Thus, upregulation of endogenous protective antioxidants might be more clinically relevant.

*In-vitro* and *in-vivo (e*.*g*. *exercise)* experiments show that shear stress to the endothelium increases endogenous antioxidants and activates endothelial nitric oxide synthase (eNOS) [9–13]. eNOS activation produces nanomolar quantities of nitric oxide (NO) which elicit endothelial dependent pulmonary and systemic vasodilation, increase blood flow, and signal increased expression of cytoprotective genes such as antioxidant enzymes [14–16]. Shear Stress induced antioxidant response has been associated with upregulation of the nuclear factor erythroid 2-related factor (Nrf2) a transcription factor that functions as the key controller of the redox homeostatic gene regulatory network.

Periodic acceleration (pGz) in humans and animal models (pigs and rodents) adds low amplitude pulses to the circulation. pGz is produced by a motorized platform that rapidly and repetitively moves the horizontally oriented body sinusoidally in a head to foot direction. Inertia of fluid as the body accelerates and decelerates adds a small amplitude pulse to the circulation that is superimposed upon the natural pulse thereby increasing pulsatile shear stress to the endothelium. Pulsatile shear stress induced by pGz releases eNOS derived NO into the circulation in amounts that are physiologically meaningful and long lasting [17, 18]. We recently found that pGz ameliorates muscle pathology in *mdx* mice [19] and reduces myocardial damage after ischemic insult [20] both pathologies are associated with elevated oxidative stress. The purpose of this study was to determine whether pGz upregulates endogenous antioxidants in hearts of normal mice and decreases oxidative stress in mice models characterized by high oxidative stress, e.g. Type 1 Diabetes and *mdx* (Duchene Muscular Dystrophy).

## Materials and Methods

### 2.1 Animal Procedures

The experimental protocol No. 14-22-A-04 was approved by the Mount Sinai Medical Center Animal Care and Use Committee and conforms to the Guide for the Care and Use of Laboratory Animals published by the National Institutes of Health (NIH Publication No. 85–23, revised 1996). Male mice C57BL/6 and dystrophic (*mdx*) (C57BL/10ScSn-*Dmd* ^*mdx*^/J) (Total n = 250) weighing between 20 and 25 g (Harlan Laboratories, Indianapolis, IN and Jackson Laboratory (Bar Harbor, Maine), were used for these experiments.

pGz was applied with a platform that moved with a linear displacement direct current motor (Model 400, 12V; APS Dynamics, Carlsbad, CA) powered by a dual mode power amplifier (Model 144, APS Dynamics), connected to a sine wave controller (Model 140–072; NIMS, Miami, FL). The controller allows control of frequency and travel distance of the platform with a read-out of acceleration from an accelerometer. The platform has a maximum weight capacity of 30 kg, operates at a frequency from 30–720 cycles/min (cpm) and achieves accelerations ranging from ±0.1 to ±14.7m/sec^2^. Optimum frequency of pGz in mice was determined as previously reported [18]. (S1 File) pGz treatment was performed on unanesthetized, restrained mice using the above described platform (1 hr. per day ƒ = 480cpm and Gz ±3.0 mt/sec^2^) for 14 consecutive days for both diabetic and *mdx* mice models and their respective controls.

### 2.2 Determination of Total Antioxidant Capacity and Antioxidant Protein Expression

Mice (n = 60) received daily pGz (1 hr per day ƒ = 480cpm and Gz ±3.0 mt/sec^2^). Myocardial tissue was harvested at baseline (BL) and twenty four hours after 1, 2, and 4 wk. of daily pGz. Protein expression of Glutathioneperoxidase-1 (GPX1), Superoxide Dismutase-1 (SOD-1, Cu-Zn SOD), and Catalase (CAT) were measured in myocardial tissue by western blot. Total Antioxidant Capacity (TC) was measured in myocardial tissue after 4 wk. of pGz or in time control mice (CONT) by ELISA (Abcam, Cambridge, MA) (S1 File).

### 2.3 Protein Expression and Genomic upregulation of Antioxidant Response Element (ARE) Transcription Factor Nrf2

We measured protein expression and genomic upregulation (RT-PCR) of Nrf2, and cytosolic to nuclear translocation of Nrf2 in mice (n = 20) hearts exposed to 4 wk. of pGz (1 hr/day) or time control (Qiagen, Valencia CA). Homogenates where fractionated to cytoplasmic and nuclear fraction by subcellular protein fractionation method (Life Technologies, Thermo Fisher Scientific, Rockford, IL.).

### 2.4 Oxidative Stress Mice Models

#### Type 1 Diabetes Model

C57BL/6J male mice (n = 20), 3 months of age, were injected with a single intraperitoneal (IP) dose of streptozotocin (STZ) (150 mg/kg body weight, in 0.1 mol/L sodium citrate buffer, pH 4.5) (Sigma–Aldrich, St. Louis, MO, USA). Aged-matched control mice received a single equal volume IP injection of sodium citrate buffer. Determination of plasma glucose 4 days after injection confirmed hyperglycemia with glucose of > 250mg/dl. Diabetic and age-matched controls mice were randomly divided into four groups of animals (n = 5 per group): i) control (CONT), ii) control pGz (CONT-pGz), iii) diabetic (Diab) and iv) diabetic-pGz (Diab-pGz) groups.

#### Duchene Muscular Dystrophy (mdx) Model

Male 12 months old C57BL/10 wild type (Wt) and dystrophic (*mdx*) (C57BL/10ScSn-*Dmd* ^*mdx*^/J) mice were obtained from Jackson Laboratory (Bar Harbor, Maine). Both *mdx* and age-matched controls mice were randomly divided into four groups (n = 4 per group): i) wild type control (Wt), ii) wild type + pGz (Wt-pGz), iii) *mdx* (mdx) and iv) *mdx*+pGz (mdx-pGz) groups.

### 2.5 Cardiomyocyte isolation

Ventricular cardiomyocytes were isolated using collagenase enzymatic digestion via retrograde perfusion. The left ventricle was dissected and minced cardiomyocytes were resuspended sequentially in various concentrations of Tyrode solution, followed by exposure to 1.5 mM Ca^2+^ for 15 min before being resuspended in normal Tyrode solution supplemented with normal Ca^2+^ concentration (1.8 mM) (S1 File).

### 2.6 Measurement of Reactive Oxygen Species

ROS activity was determined in isolated cardiomyocytes using the fluorescent method of chloromethyl-2,7-dichlorodihydrofluorescein diacetate (CM-DCFDA Molecular Probes, OR, USA). (S1 File)

### 2.7 Protein Expression and Phosphorylation

Protein extraction, RNA and subcellular fractionations where performed as previously described. Protein analysis was performed using Western Blot method and RT-PCR blots visualized by enhanced chemifluorescence (S1 File).

### 2.8 Euthanasia

After completion of each of the experimental protocols animals were euthanized by a method approved by the American Veterinary Medical Association Guidelines on Euthanasia (S1 File).

### 2.9 Statistical Analysis

Statistical analysis of the data was accomplished with software running on a personal computer Statistica (StatSoft, Tulsa, OK). Results were expressed as mean percent (%) to baseline with SD or Mean ± SD. and comparison of means with 95% confidence levels was carried out using analysis of variance (ANOVA) followed by *post hoc* analysis with the Newman-Keuls test. Statistical significance was established at p<0.05. Sample size was calculated using Statistica based on power analysis with α = 0.05 and power 0.80.

## Results

### 3.1 Effects of pGz frequency, on Mean Aortic Pressure, Heart Rate and a/b in normal mice

pGz at all frequencies from 360 to 600 cpm added pulses visible on the aortic pulse waveform of 1–3 mmHg as a function of frequency. These added pulses occurred at peaks and troughs of acceleration of the motion platform. The descent of the dicrotic notch pressure wave (a/b) cycled downward to upward over periods ranging from 2 to 5 seconds but the overall downward descent was maintained throughout the pGz period. The descent of the dicrotic notch was greatest at a pGz frequency of 480cpm, decreasing by more than 90% of BL values.

There were no significant changes from BL in mean heart rate with application of pGz at all frequencies. pGz decreased mean arterial pressure (MAP) within five minutes of its application but the a/b ratio increased about 25 to 50% from baseline within 2 seconds. MAP progressively decreased during 30 minutes after pGz. pGz administration decreased MAP from baseline of 67 ±3 to 59 ±4, 52 ±2, 53±5 mmHg after 30 min of pGz at 360, 480, 600 cpm respectively (*p*<0.05 Bl vs. pGz) (S1 File).

### 3.2 Time Course of Endothelial Nitric Oxide Synthase, Akt and Expression and Phosphorylation after pGz

One, 4 and 8 days of pGz increased eNOS, p-eNOS, and ratio of p-Akt/Akt. Phosphorylation of eNOS and Akt was most pronounced after 1 day of pGz. Protein and genomic expression of eNOS were most pronounced after 8 days of pGz. After 8 days of pGz, upregulation of eNOS peaked at 48 hrs after the last pGz session and gradually declined as a function of elapsed time from last pGz session. In a Duchene Muscular Dystrophy mouse model (*mdx*) eNOS and p-eNOS were significantly decreased compared to Wt controls. Four weeks of pGz in *mdx* significantly restored both eNOS and p-eNOS to close to Wt levels (S1 File).

### 3.3 pGz Induces Antioxidant Expression and Increases Antioxidant Capacity

**Fig 1:**
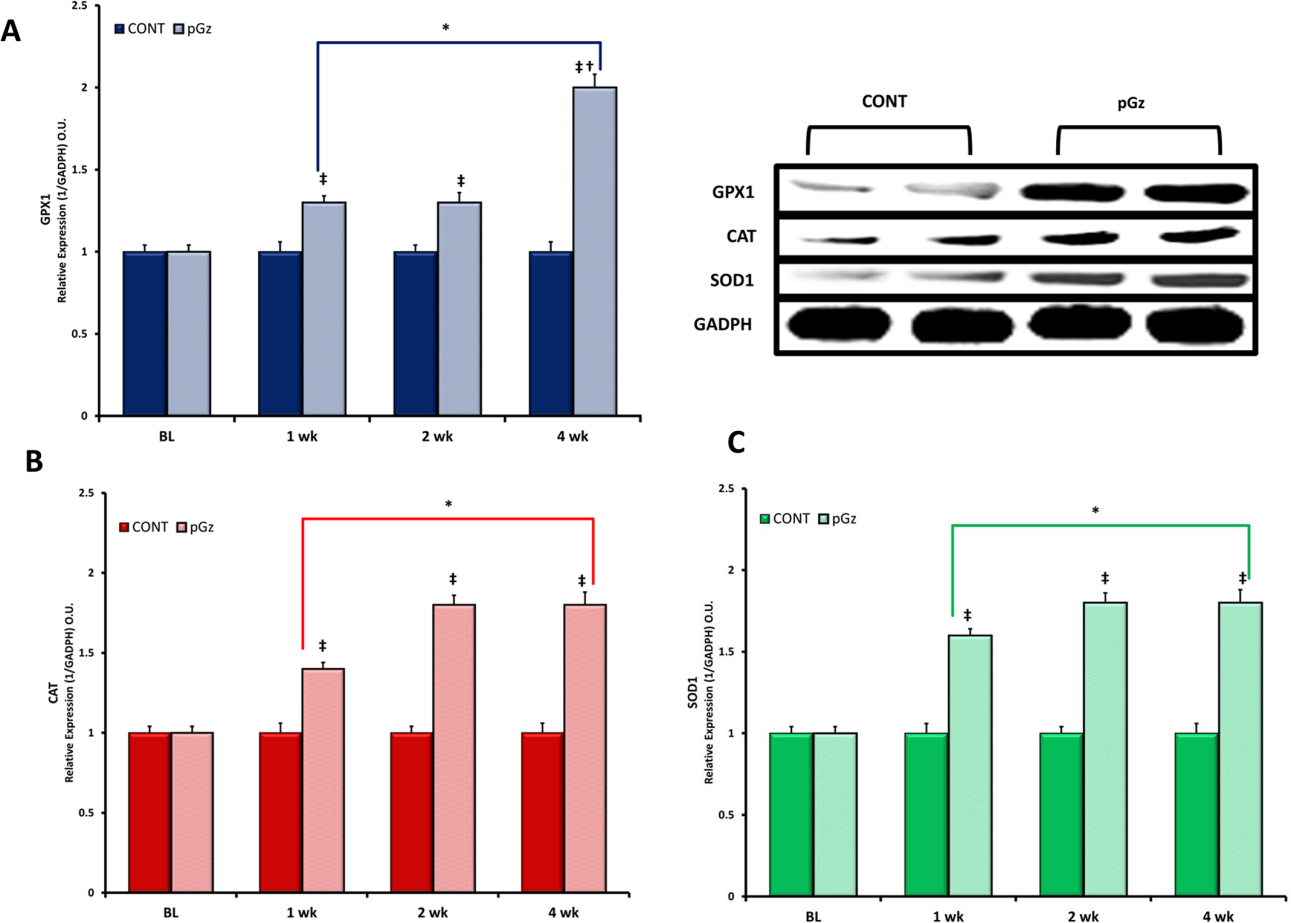
Antioxidant Protein Expression is Induced by pGz. This figure depicts the effects of 1, 2, and 4 wk. of daily pGz in normal mice on the expressions of **(A).** Glutathioneperoxidase-1 (GPX1), **(B).** Catalase (CAT), and **(C)**. Superoxide Dismutase 1 (SOD1). The relative protein expressions of GPX1, CAT and SOD1 over time compared to time of CONT values showed that pGz significantly increased GPX1, CAT, and SOD1 expression at 1, 2 and 4 wk compared to time CONT (^**‡**^p < 0.01 1, 2, and 4 wk. pGz vs. CONT). One, 2, and 4 wk. of pGz significantly increased expression of GPX1, CAT, and SOD1 compared to baseline (BL) values for both CONT and pGz groups (* p< 0.01 1,2, and 4 wk. vs. BL values). Representative Immunoblots of protein expression of GPX1, CAT, SOD1 and protein quantity loading Glyceraldehyde 3-phosphate dehydrogenase (GADPH) after 4 wk. of pGz, for CONT and pGz groups showing significant expression of these compared to time CONT.

**Fig 2:**
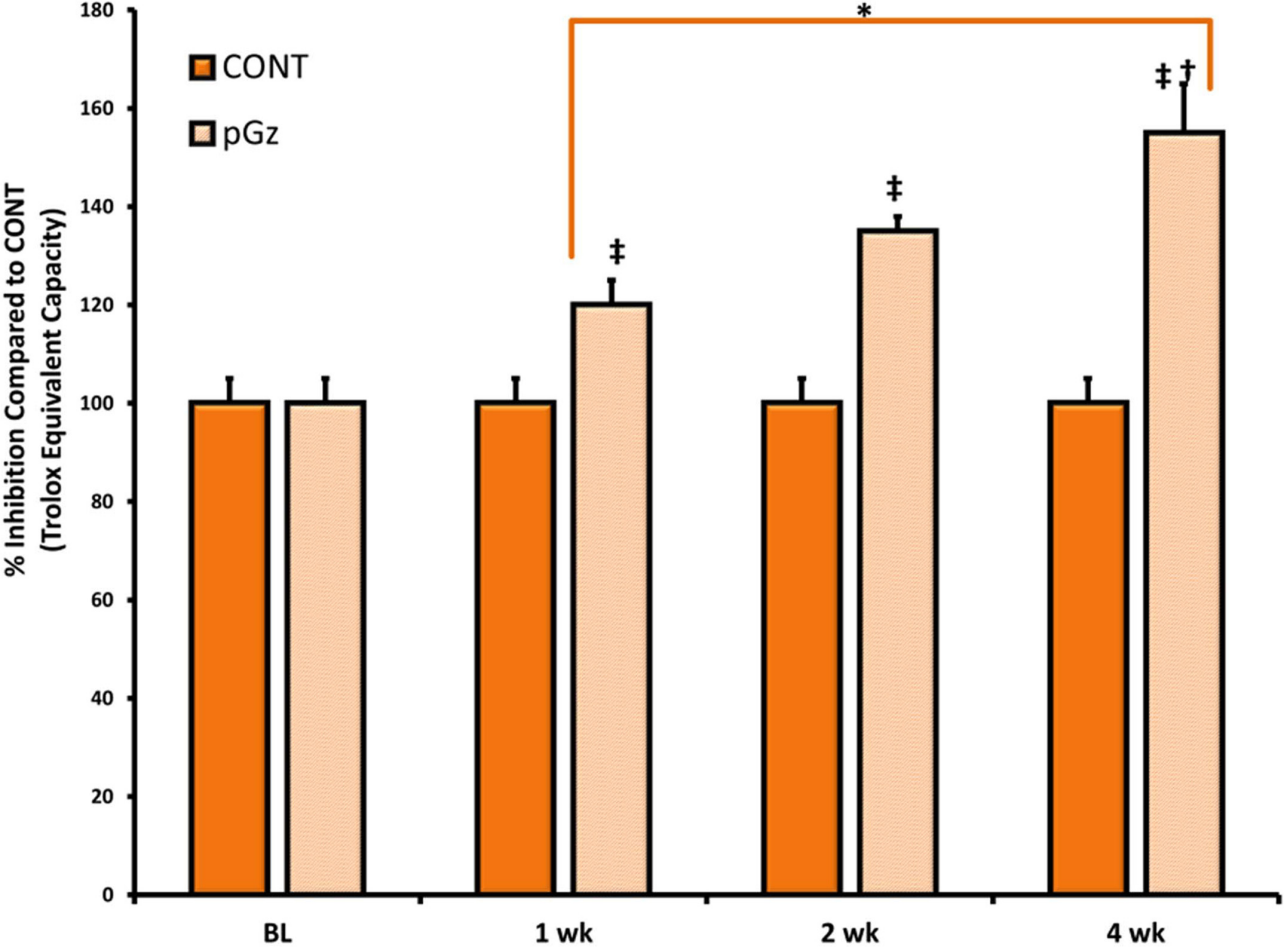
pGz Increases Total Antioxidant Capacity. Total antioxidant capacity (Trolox Equivalent Capacity) was expressed as % Inhibition of control values, measured at baseline (BL) for CONT and pGz groups, and after 1, 2 and 4 wk. of pGz or time CONT. One, 2 and 4 wk. of daily pGz significantly increased antioxidant capacity compared to time CONT (^**‡**^p < 0.01 1, 2 and 4 wk. vs. time CONT). One, 2, and 4 weeks of pGz also significantly increased total antioxidant capacity compared to baseline (BL)values for both CONT and pGz (* p< 0.01 1,2 and 4 wk. vs. BL values for CONT and pGz groups). Optical Units = O.U.

**Fig 3:**
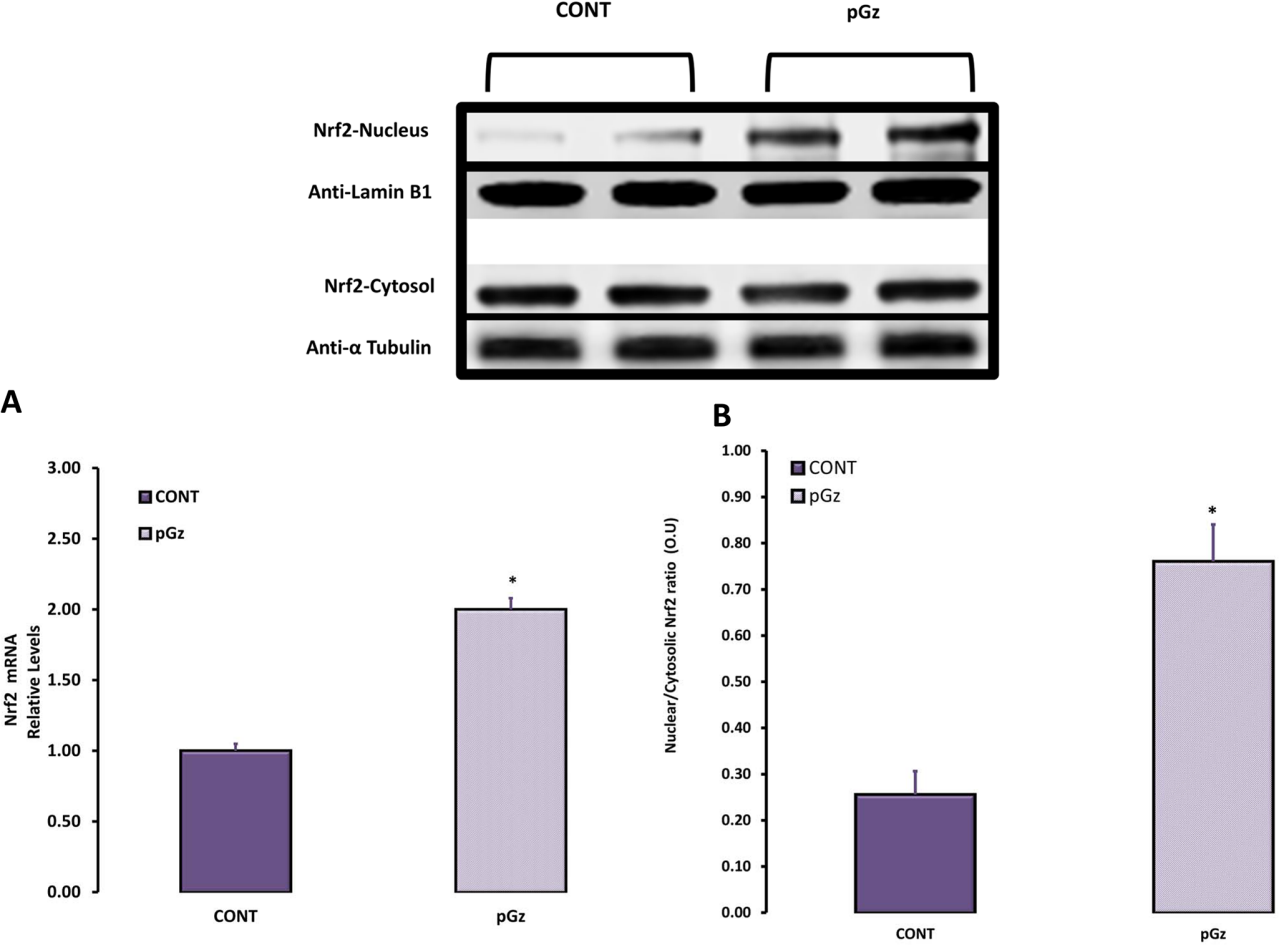
The Expression and Translocation of Nrf2 Transcription Factor after pGz Treatment. This figure depicts the **e**xpression and translocation of Nrf2 transcription factor after 4 wk of daily pGz or no pGz (CONT) **(A)**. pGz significantly increased upregulation of the Antioxidant Response Element transcription factor Nrf2 compared to CONT (*p< 0.01) **(B**). Translocation of Nrf2 is expressed as the ratio of Nrf2 in nucleus and cytosol. pGz also significantly increased Nrf2 translocation to nucleus compared to CONT (*p< 0.01). Representative Immunoblots of Nrf2 in nucleus and cytosol with respective protein loading controls Anti-Laminin B1 (nucleus) and Anti-α Tubulin (cytosol) in CONT and pGz groups showed increased protein expression of Nrf2 in nucleus of pGz treated mice. Optical Units = O.U.

Induction of antioxidant enzymes by pGz was measured after 1, 2 and 4 wk. of daily pGz. Peak effect of enzymatic expression and total antioxidant capacity was seen after 4 wk. of pGz for GPX1, CAT, SOD1 (Figs 1 and 2). After 4 wk. of pGz, protein expression and upregulation of the antioxidant response element, transcription factor Nrf2, was significantly increased. Furthermore, pGz induced Nrf2 translocation from cytosol to nucleus (Fig 3).

### 3.4 Effects of pGz in Models of Oxidative Stress

**Fig 4:**
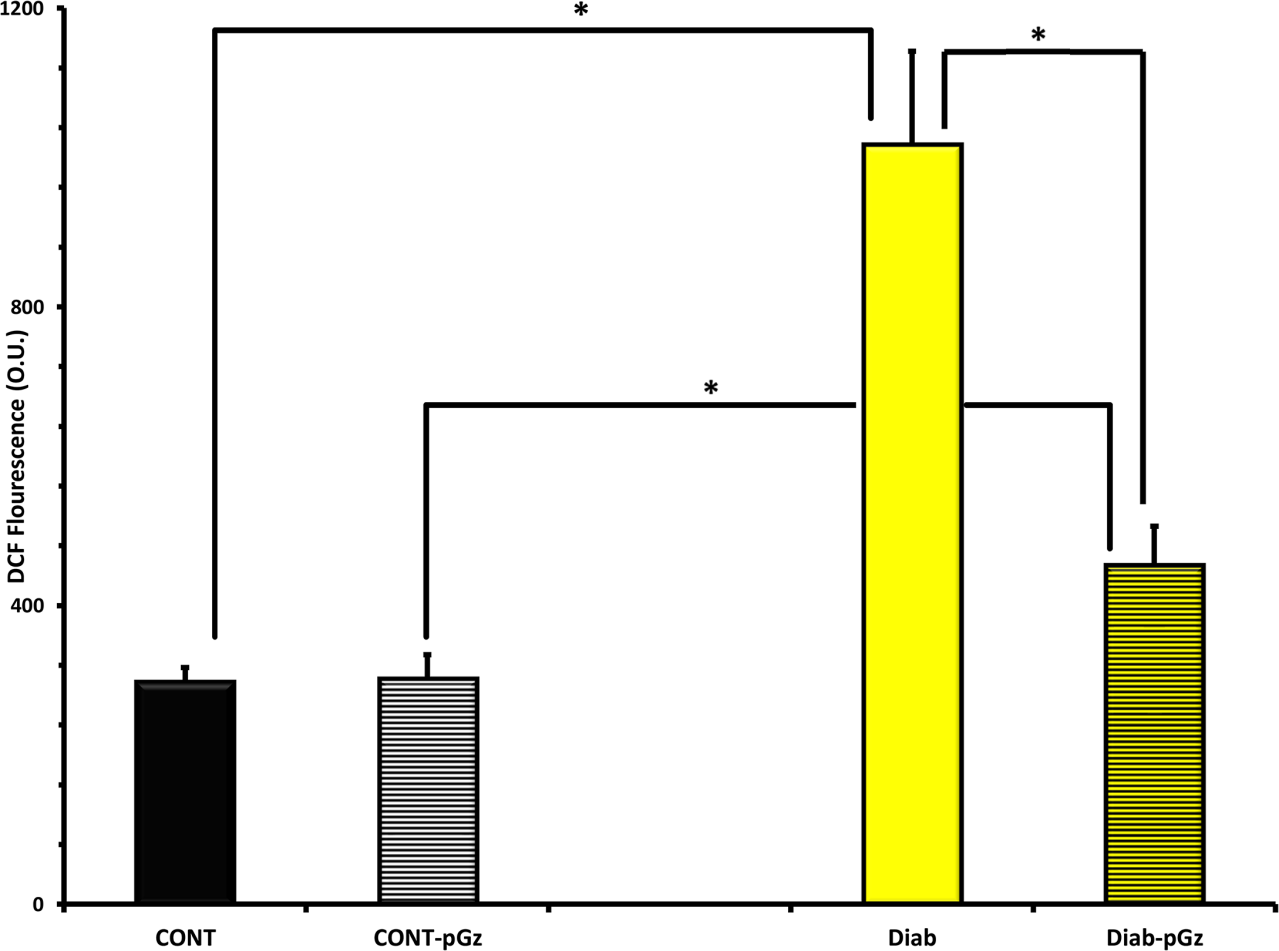
The Effects of pGz treatment on Diabetes Induced Oxidative Stress in Cardiomyocytes. The effects of pGz treatment for 14 days on ROS in cardiomyocytes in Control (CONT-pGz) and Diabetic mice (Diab-pGz) and their respective controls without pGz treatment in non diabetic (CONT) and diabetic (Diab). ROS was measured using the method of DCF fluorescence. Diabetes significantly increased ROS in cardiomyocytes (*p < 0.001 CONT vs. Diab). Treatment with pGz significantly reduced the diabetes induced increase in ROS (*p < 0.001 Diab vs. Diab-pGz and CONT-pGz vs Diab-pGz). Optical Units = O.U.

**Fig 5:**
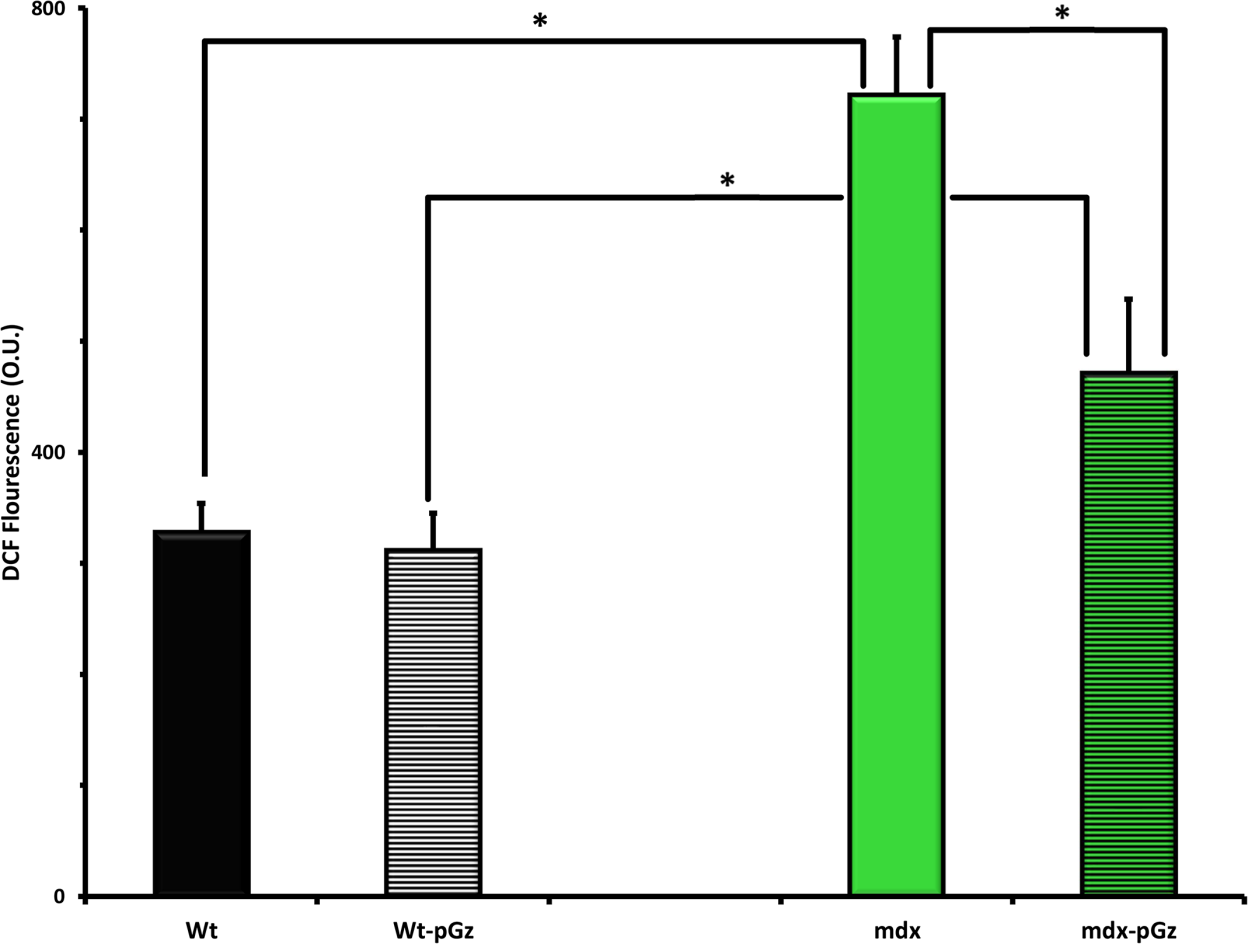
The Effects of pGz treatment on Duchene Muscular Dystrophy Induced Oxidative Stress in Cardiomyocytes. Twelve month of age *mdx* mice had a significant increase in oxidative stress in cardiomyocytes compared to age matched wild type (Wt) controls (*p < 0.001). Treatment with pGz (mdx-pGz) for 14 days significantly reduced the levels of ROS measured by DCF fluorescence (*p<0.001) Optical Units = O.U.

In order to study whether or not pGz reduces oxidative stress, we studied the effects of pGz on ROS production in two models of cardiovascular disease associated with elevated oxidative stress, type 1 diabetes and *mdx*. Type 1 Diabetes induces elevated cardiac oxidative stress as demonstrated in cardiomyocytes, ROS was 3.4 fold higher in diabetic mice than Wt (1017±28.0 to 298±4.3 O.U. (p< 0.001). Fourteen days of daily pGz application significantly reduced ROS from 1017±28.0 to 454 ±11.7 O.U, (p < 0.001) (Fig 4). The 12 mos age *mdx* mouse model of Duchene Muscular Dystrophy, is also characterized by significant amount of oxidative stress in heart with levels of ROS of 722±11.7 compared to 328±7.1 O.U in Wt, (p < 0.001), pGz also significantly attenuated oxidative stress in the *mdx* model from 722±11.7 to 471±15.0 O.U., (p < 0.001) (Fig 5).

## Discussion

The present study demonstrates that one hour daily treatments of pGz to normal mice increased expression and phosphorylation of eNOS in the heart as a function of the number of treatments over time. Application of pGz through increased pulsatile shear stress activates eNOS through phosphorylation and genomic upregulation in cells and animal models in part mediated via the Akt/PI3K pathway [21] [18]. In the *mdx* model where eNOS and p-eNOS are decreased compared Wt, pGz also restored both. Further, we have also shown in other animal models in which eNOS and p-eNOS are also decreased such as whole body ischemia reperfusion injury [22], and focal myocardial ischemia [23] that pGz also significantly restores these. The clinical importance of increasing and activating eNOS has been reviewed by others [24–27].

Endogenous antioxidant enzymatic expressions and activity were also increased by pGz. Various methods to increase shear stress have been used to augment antioxidant levels. *In-vitro* studies have shown that pulsatile shear stress on the vascular endothelium increases Cu/Zn SOD (SOD1) and GPX1 [9, 28–31]. Exercise is an intervention which also increases shear stress. Aging mice studies have shown that chronic moderate treadmill exercise produces a mild effect at increasing the activities of Mn-SOD, SOD1, and catalase in brain, heart, liver, and kidney of mice exercised for 24 to 50 wk. After 24 weeks of aerobic exercise, antioxidant enzyme increased by 15–20% [13]. Additionally, the beneficial effects of exercise in diseased animal models have in part been shown to be related to augmentation of antioxidant defenses [32, 33]. Exercise may or may not be beneficial in *mdx* mice (model of Duchene Muscular Dystrophy, DMD). In this model, *mdx* mice running for 4 or 10 weeks accelerates ventricular dilatation and fibrosis [34–36] whereas another study showed that voluntary wheel running for one yr. produced positive exercise–induced remodeling in the heart [37].

Diabetic cardiomyopathy is a well-known complication of diabetes. Diabetic cardiomyopathy is characterized by early diastolic dysfunction and adverse structural remodeling leading to heart failure (HF). Pre-clinical studies confirm a major causal role for elevated myocardial ROS generation in diabetic cardiomyopathy [38]. In a type 1 diabetic rat model, 9 weeks of low intensity exercise provided protection from cardiomyopathy in part via augmentation of the antioxidant capacity [39]. Additionally, augmentation of extracellular SOD using a transgenic mouse model with type 1 diabetes protects from cardiac hypertrophy, fibrosis and dysfunction [40]. Thus, reduction of oxidative stress and augmentation of antioxidants could prevent/ameliorate the development and progression of diabetic cardiomyopathy [41, 42]. In the current study pGz increased antioxidant capacity and reduced ROS generation in diabetic cardiomyocytes, thus making it an attractive therapeutic modality.

A possible mechanism whereby pGz increases antioxidant capacity may involve the antioxidant response element (ARE) transcription factor Nrf2 which serves as the key controller of the redox homeostatic gene regulatory network. This factor was upregulated after pGz and translocated to the nucleus site of activity. Nrf2 regulates expression of genes containing antioxidant response element in their promoters e.g. heme oxygenase-1(*HO-1*), NAD(P)H quinone oxidoreductase 1(*NQO1*) [12, 43–49]. The Nrf2/Keap1/ARE signaling pathway has been used as a pharmacological therapeutic target [50, 51]. Nrf2 activity is reduced in diabetic cardiomyopathy and its activation has been shown to protect from diabetic cardiomyopathy[52]. Similarly, Nrf2 induction via sulforaphane has also been shown to improve muscle function and pathology in *mdx* [53].

There are limitations to the present work. We did not explore exposure to pGz for longer than 4 wk. and beneficial effects beyond this time period can only be speculated upon. We also did not address eNOS uncoupling in this study, the latter is highly unlikely in the setting of endogenous physiological production of NO in non-diseased mice.

Oxidative stress and vascular inflammation play a pivotal role in cardiovascular health and particularly in the aging human population [54]. The therapeutic value of enhancing antioxidant capacity for cardiovascular protection has been reviewed by others [51, 54, 55]. pGz is a noninvasive simple intervention, which does not require subject cooperation, and can be performed in persons with physical and or cognitive impairment. Since pGz has been safely used in humans with various cardiovascular diseases, our findings in these animal models are very clinically relevant [56–60]. In addition to the well-known salutary effects produced by activation and upregulation of eNOS, pGz also increases endogenous antioxidant capacity and reduces indices of oxidative stress, which have profound therapeutic potential.

## Supporting Information

S1 File This file contains additional information on Materials & Methods used in this study and expanded Results. The Materials and Methods include; a) method for the determination of optimum endothelial vasodilatation by pGz in mice, b) methods for the determination of the time course of protein expression and phosphorylation of eNOS and Akt, c) methods for the determination of ROS in cardiomyocytes, d) supplemental description for methods for protein expression and phosphorylation, e) method for animal euthanasia. The Results section includes; a) representative tracing of the aortic pulse waveform in mice and the effects of pGz of varying frequency on the position of the dicrotic notch, b) figures for the effect of pGz frequency on mean arterial blood pressure and the change in a/b ratio, c) figures on the effects of duration of pGz on protein expression of eNOS and the ratio of p-eNOS/eNOS, d) figures on the effects of pGz on genomic upregulation of eNOS, e) figures on the effects of duration of pGz on protein expression of Akt and p-Akt/Akt, f) figures on the effect of 4 weeks of pGz on eNOS and p-eNOS protein expression in control (wt) and Duchenne Muscular Dystrophy (mdx) mouse model.
(DOCX)
