# Supplementary material for: Antioxidant Properties of Whole Body Periodic Acceleration (pGz)
Source: PLoS One. 2015 Jul 2;10(7):e0131392. doi: 10.1371/journal.pone.0131392 (PMC4489838; doi:10.1371/journal.pone.0131392)
Supplement: S1 File — The Materials and Methods include; a) method for the determination of optimum endothelial vasodilatation by pGz in mice, b) methods for the determination of the time course of protein expression and phosphorylation of eNOS and Akt, c) methods for the determination of ROS in cardiomyocytes, d) supplemental description for methods for protein expression and phosphorylation, e) method for animal euthanasia. The Results section includes; a) representative tracing of the aortic pulse waveform in mice and the effects of pGz of varying frequency on the position of the dicrotic notch, b) figures for the effect of pGz frequency on mean arterial blood pressure and the change in a/b ratio, c) figures on the effects of duration of pGz on protein expression of eNOS and the ratio of p-eNOS/eNOS, d) figures on the effects of pGz on genomic upregulation of eNOS, e) figures on the effects of duration of pGz on protein expression of Akt and p-Akt/Akt, f) figures on the effect of 4 weeks of pGz on eNOS and p-eNOS protein expression in control (wt) and Duchenne Muscular Dystrophy (mdx) mouse model. (DOCX) [file pone.0131392.s001.docx]

S1 File

Supporting Information

Materials & Methods

***Determination of Optimum Endothelial Vasodilatation by pGz.***

Mice (n=20) were anesthetized with IP Ketamine (60 mg/kg)/ Xylazine (10 mg/kg) until a surgical plane of anesthesia was obtained and supplemented throughout the experiment along with support doses as necessary. A tracheotomy was performed and an endotracheal tube inserted. A size 1F **Millar Mikro-Tip® pressure catheter was inserted into t**he right carotid artery to measure ascending aortic pressure and dicrotic notch position (Millar Instruments, Houston, TX). The a/b ratio was computed from the aortic pressure pulse where ‘a’ is the height of the pulse amplitude divided by ’b’, the height of the dicrotic notch above the end-diastolic level. An increase of the a/b ratio signifies vasodilatation and a decrease vasoconstriction. The animals were maintained at 38 °C with a thermostatically controlled warming pad. The animals were placed on the motion platform in the supine position with the front and hind legs taped securely to the table and mice body closely coupled to the platform. The tracheotomy tube was connected to a small animal mechanical ventilator delivering FiO_2_ 0.21 (model CIV-101, Columbus Instruments, Columbus, OH).

The animals were secured to the platform and continuously monitored during application of pGz. A Lead II electrocardiogram (ECG) was continuously monitored from a standard three lead configuration. Mice were subjected to 30 minutes of pGz at frequencies of 360, 480 and 600 cpm with peak acceleration amplitude of ± 3.0 mt/sec^2^. The aortic pressure and electrocardiogram (ECG) were recorded at baseline, and continuously during pGz. The individual a/b ratios were measured for 30 seconds at the end of each period of baseline (BL) and pGz and a mean value computed.. The dicrotic notch position was readily identified on the aortic pressure wave. A twenty-beat ensemble average was applied to the aortic pressure wave to obtain the mean a/b ratio. In addition, the following where computed from the aortic pressure catheter signal; mean heart rate and mean aortic pressure. The analog signals from the transducers, accelerometer, ECG, and aortic catheter were continuously recorded on a data acquisition processor (Power Lab, AD Instruments, CO) at a rate of 1000 samples per second, as previously described by our laboratory [1,2].

***Time Course of eNOS and Akt Expression and Phosphorylation.***

Awake unanesthesized mice (n=60) were acclimated for 3 days in a mice restrainer (Kent Scientific, Torrington, CN) to achieve 1 hr total duration of restraint. Mice were randomized to receive pGz at the optimum frequency of 480 cpm, and Gz ± 3.0 mt/sec^2^ (1 hr/day) for 1, 4, or 8, days (pGz ) or serve as time control (CONT). Twenty four hours after the last pGz session or control the hearts were harvested. An additional group of acclimated mice (n=50) were also randomized to pGz or time control (CONT) for 8 days and hearts harvested at 4, 12, 24, 48, 72 hrs after the last session in order to determine the time course of optimum eNOS transcription .

***Determination of ROS in Mice Models***

*Cardiomyocyte isolation*

Ventricular cardiomyocytes were isolated using collagenase enzymatic digestion via retrograde perfusion. Mice were anesthetized via intraperitoneal (i.p.) injection of ketamine-xylazine mixture (80 and 10 mg/kg), and then the heart was excised and placed in a dish of oxygenated, nominally Tyrode Ca^2+^-free solution (no Ca^2+^ was added). The aorta was cannulated and connected to a perfusion system. The heart was retrograde perfused with Tyrode Ca^2+^-free solution at a rate of 2.2 ml/min for 10 min and then with Tyrode Ca^2+^-free solution supplemented with 150 U/ml of type II collagenase (Worthington) and 0.02% protease type XIV (Sigma) for 5 min, followed by a 5-min washout with Tyrode enzyme-free solution supplemented with 0.2 mM Ca^2+^. The temperature of all perfusates was maintained at 37°C and all solutions were continuously bubbled with 100% oxygen. The left ventricle was dissected, minced and placed in a small Erlenmeyer flask containing Tyrode solution supplemented with 0.25 mM Ca^2+^ for agitation in a 37°C water bath for 5 min. The cardiomyocytes were resuspended sequentially in Tyrode solution supplemented with 0.5 mM, and 1 mM Ca^2+^ concentration for 5 min in each solution. They were then exposed to 1.5 mM Ca^2+^ for 15 min before being resuspended in normal Tyrode solution supplemented with normal Ca^2+^ concentration (1.8 mM). All cardiomyocytes used in this study had well-define striatal spacing and did not spontaneously contract when they were perfused with Tyrode solution supplemented with normal Ca^2+^ concentration

#### *Measurement of reactive oxygen species*

To determine intracellular ROS activity cardiomyocytes were loaded with 10μM chloromethyl-2,7-dichlorodihydrofluorescein diacetate (CM-DCFDA Molecular Probes, OR, USA) for 30 min. CM-H2DCFDA is a cell-permeant indicator for ROS in cells, that is non-fluorescent until removal of the acetate groups by intracellular esterases and oxidation within the cell. Once CM-H2DCFDA is hydrolyzed to DCFH, it can be oxidized by hydrogen peroxide or peroxynitrite to form highly fluorescent compound DCFCM-DCF. Fluorescence measurements (492-495-ex and 517–527-em nm) were conducted on cardiomyocytes at room temperature. Images were captured on a Zeiss Axiovert 135M microscope (Carl Zeiss MicroImaging, Thornwood, New York).

*Protein Expression and Phosphorylation*

As previously describe by our laboratory, the entire heart was minced and homogenized at 4 ^o^C followed by one step total protein extraction with an Extraction Buffer System (Invitrogen Corporation**,** Carlsbad, CA) or subcellular fractionation of tissue homogenates was achieved using NE-PER™ Nuclear and Cytoplasmic Extraction Reagents ( Life Technologies, Thermo Fisher Scientific, Rockford, IL.) according to the manufacturer’s protocol.

The extracted cardiac protein was measured by the BCA Protein Assay (Thermo Fisher Scientific, Waltham, MA) on a SpectraMax Plate Reader (Molecular Devices, Sunnyvale, CA). Individual proteins were then analyzed by Western blot. Equal amounts of total protein were separated on 4-12% NuPAGE Novex Bis-Tris SDS-PAGE Gels **(**Invitrogen Corporation, Carlsbad, CA**)** and transferred to Immobilon-FL PVDF membrane (Millipore Corporation, Billlerica, MA). The PVDF membrane was treated with a blocking agent (GE Healthcare Bio-Sciences Corporation, Piscataway, NJ) and probed with primary, fluorescein-linked secondary antibodies as well as anti-fluorescein alkaline phosphatase conjugate.The following primary antibodies were used; eNOS, p-eNOS ^(Ser1177),^ Akt (total Akt-1, 2, 3), p-Akt ^(Thr308)^, (Santa Cruz Biotechnology, Inc. Santa Cruz, CA.) Glutathione Peroxidase 1(GPX1), Catalase (CAT), Superoxide Dismutase 1(SOD1), nuclear factor erythroid 2-related factor (Nrf2), (Abcam, Cambridge, MA). Individual Protein loading controls used are: Glyceraldehyde-3-phosphate dehydrogenase (GAPDH) and Actin for whole tissue, Alpha-Tubulin (Tubulin) for cytosolic fraction, Laminin B1(Laminin) for nuclear fraction (Abcam, Cambridge, MA). Blots were visualized by Enhanced Chemifluorescence (ECF) (GE Healthcare Bio-Sciences Corporation, Piscataway, NJ) on Storm 860 Imaging System (GE Healthcare Bio-Sciences Corporation, Piscataway, NJ). The Storm 860 Imaging System exhibits a linear response to fluorescent signal intensities and protein levels were quantified using ImageQuant software (GE Healthcare Bio-Sciences Corporation, Piscataway, NJ). RNA was extracted using TRIReagent (Sigma, St. Louis, MO). One step RT-PCR was performed using a commercially available kit (Qiagen, Valencia, CA). PCR products were separated on 1% agarose gel, visualized under UV, and quantified using Image J 1.36b (NIH, Bethesda, MD) as previously described [2-4].

***Euthanasia***

After completion of each of the experimental protocols, animals were euthanized by a dose of Ketamine 90mg/kg and Xylazine 25mg/kg, followed by pentobarbital 100mg/kg IP, until absence of corneal and pedal reflex, and no electrical activity on ECG, and decapitation via guillotine, a method approved by the American Veterinary Medical Association Guidelines on Euthanasia. Perfusion and or organ harvesting was performed as per protocol [5-7].

RESULTS:

**
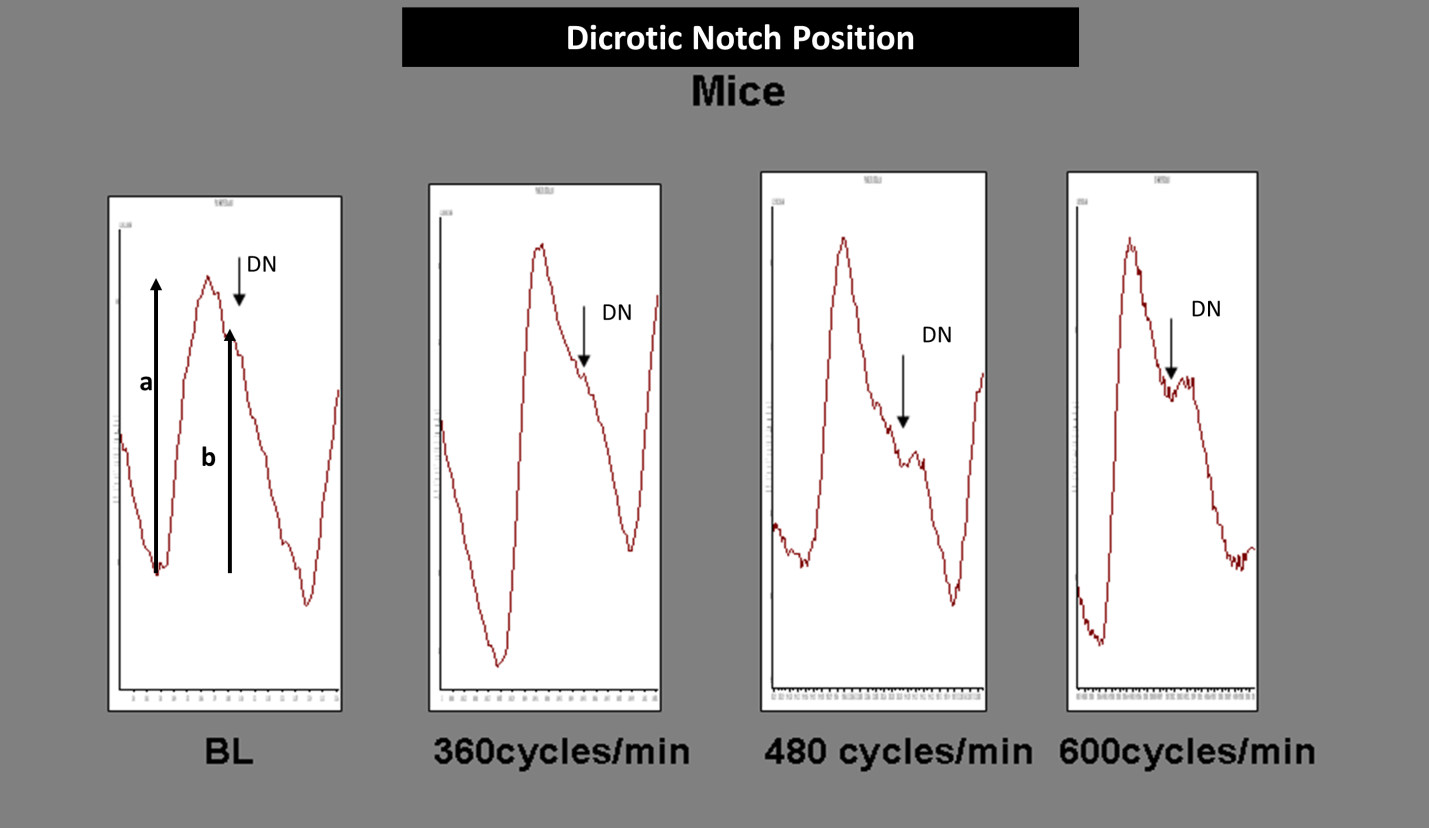
**

Representative tracing of the aortic pulse waveform at baseline (BL) and after pGz of 360, 480, 600 cycles per min(CPM). The amplitudes of the “a” and “b” segments of pressure waves are shown for baseline (BL). Dicrotic notch position (DN) (**↓**) for each pulse waveform. pGz produced decreased a downward displacement of DN at all frequencies. Maximal DN displacement occurred at 480cycles per min.

Representative tracing of the aortic pulse waveform at baseline (BL) and after pGz of 360, 480, 600 cycles per min(CPM). The amplitudes of the “a” and “b” segments of pressure waves are shown for baseline (BL). Dicrotic notch position (DN) (**↓**) for each pulse waveform. pGz produced decreased a downward displacement of DN at all frequencies. Maximal DN displacement occurred at 480cycles per min.


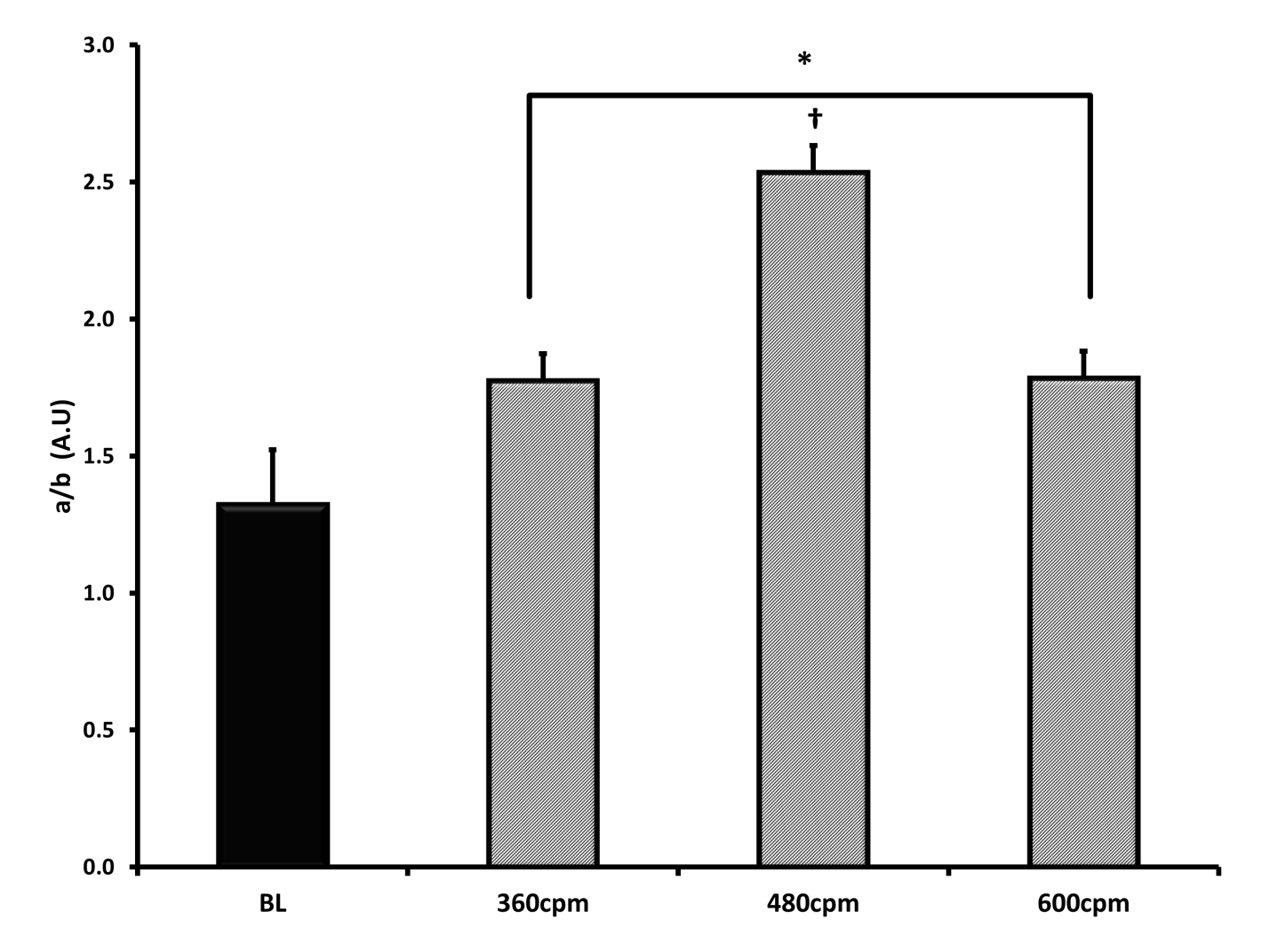


**Change in a/b Ratio as a Function of pGz Frequency**

Change of a/b ratio in response to pGz, as a percent change from BL value. Frequency of 480 cpm produced the largest increase in a/b as a percent of BL (* p< 0.001).

**
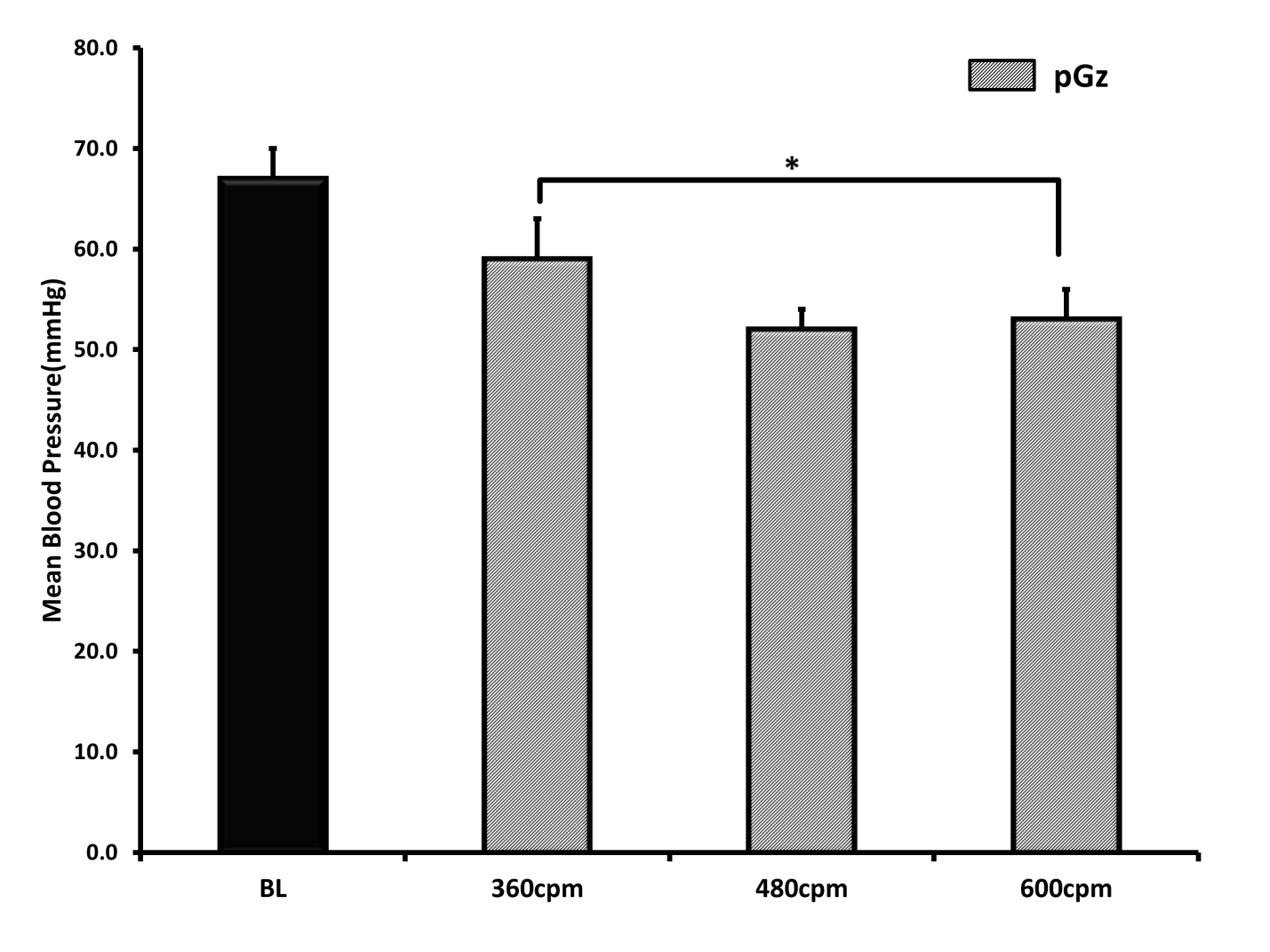
**

**Changes in Mean Arterial Pressures with pGz**

Mean arterial blood pressure at BL,360, 480, and 600 cycles per min (cpm). Significant decrease in blood pressure at all frequencies compared to BL (*p< 0.01).

**
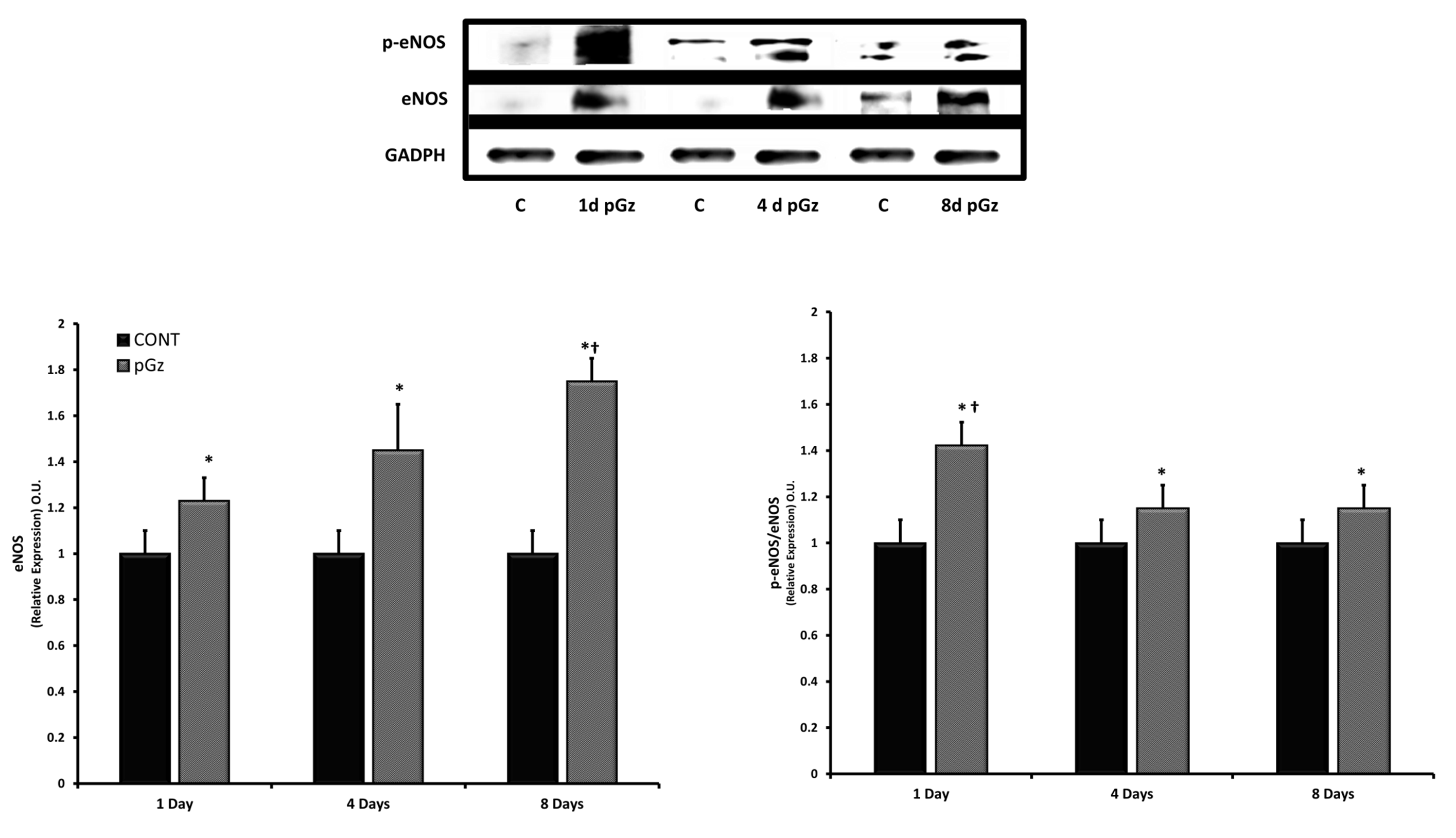
**

**pGz Induces Protein Expression and Phosphorylation of eNOS.**

The effects of 1, 4, 8 days of pGz on protein expression of eNOS and ratio p-eNOS/eNOS relative to time control (CONT) values. pGz increased eNOS expression at all time periods, with the largest effect after 8 days of pGz (* p< 0.01 1 ,4 and 8 days vs. time CONT and † p< 0.01 8 vs. 1 and 4 days). The ratio of p-eNOS/eNOS was increased after 1,4,and 8 days of pGz compared to time CONT (* p< 0.01 1 ,4 and 8 days vs. time CONT) with the largest increase after 1 day († p< 0.01 8 vs. 1 and 4 days). Representative Immunoblots of protein expression of eNOS, p-eNOS and protein loading quantity for Glyceraldehyde 3-phosphate dehydrogenase (GADPH), for CONT and days 1,4,8 (c, 1dpGz, 4dpGz, 8dpGz). Optical Units=O.U.


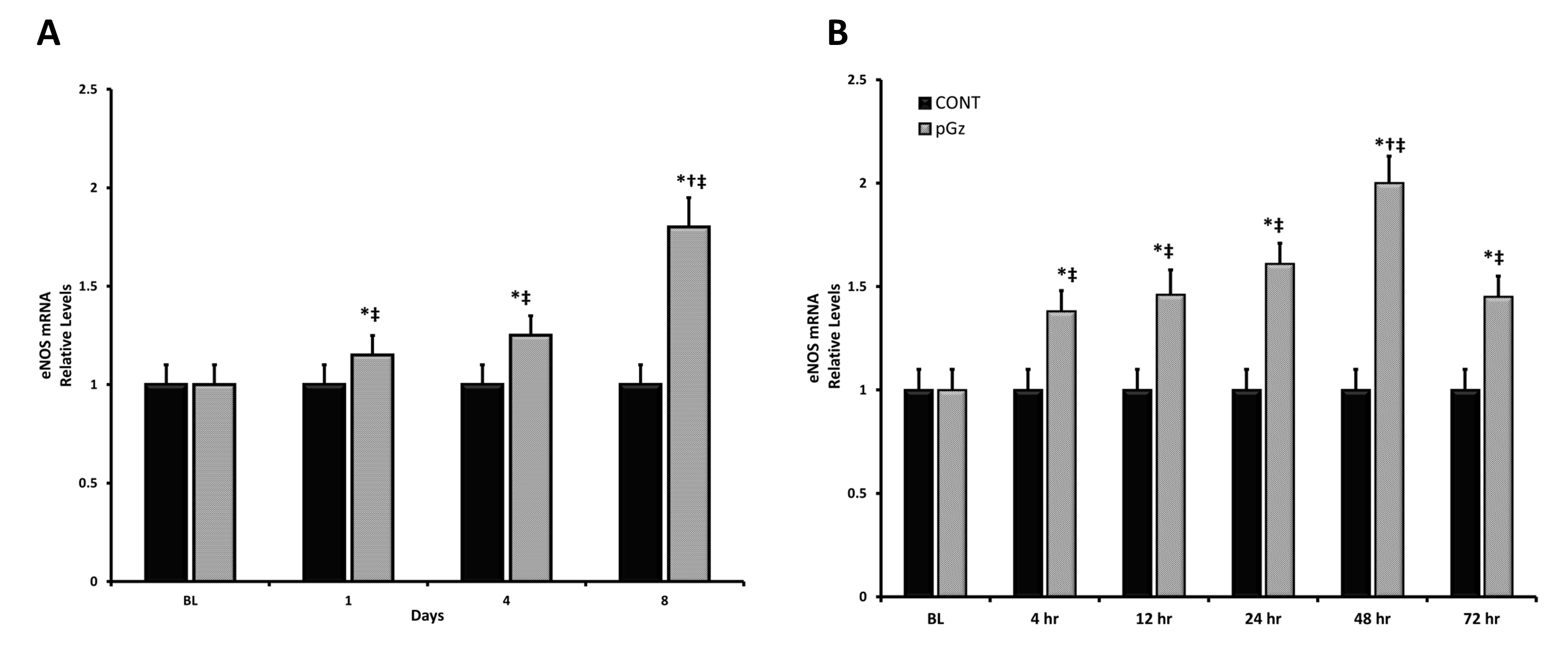


**pGz Induces Upregulation of eNOS**

1. The effects of 1,4, and 8 days of pGz on eNOS upregulation (mRNA). pGz increased eNOS mRNA at all time periods, with the largest effect after 8 days of pGz (* p< 0.01 1 ,4,and 8 days vs. CONT and ^†^ p< 0.01 8 days vs. 1 and 4 days, **^‡^** p< 0.01 1,4, and 8 days vs. Baseline(BL) CONT and pGz)
2. pGz was performed for 8 consecutive days (maximal upregulation, *f*=480cpm 1hr per day) or time CONT, and the increase and decay of eNOS mRNA was measured overtime. After 8 days of pGz eNOS mRNA was significantly increase at 4, 8,12,24,48 and 72 hr. after cessation of pGz, compared to baseline (BL) CONT and pGz values and compared to time CONT. Peak eNOS expression occurred at 48 hrs after cessation of pGz, and declined after 72hr. (* p< 0.01 4,8,12,24,48,and 72 hr. vs. time CONT , **^‡^** p < 0.01 4,8,12,24,48 and 72 hr. vs. BL CONT and pGz values, and † p< 0.01 48 hr. vs. 4,8,12,24,72 hr.)


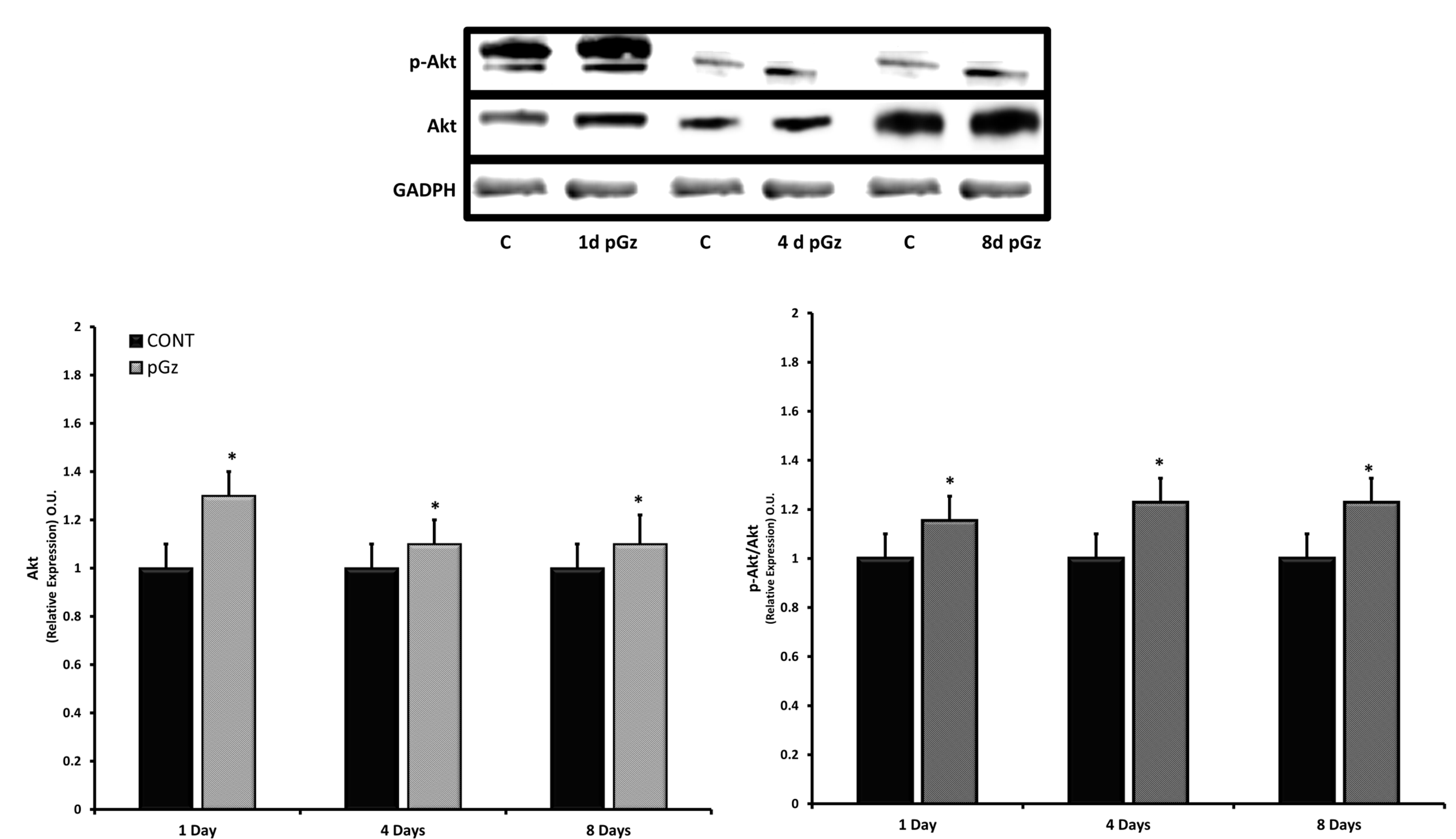


**Protein Expression and Phosphorylation of Akt Induced by pGz**

The effects of 1, 4, and 8 days of pGz on protein expression of Akt and ratio of p-Akt/Akt relative to time CONT values. pGz increased Akt expression at all time periods (* p< 0.01 1 ,4,and 8 vs. time CONT ). The ratio of p-Akt/Akt was also increased after 1,4 and 8 days of pGz compared to time CONT (* p< 0.01 1 ,4, and 8 days vs. time CONT). Representative Immunoblots of protein expression of Akt, p-Akt and protein quantity loading Glyceraldehyde 3-phosphate dehydrogenase (GADPH), for CONT and days 1,4,8 (c, 1dpGz, 4dpGz, 8dpGz). Optical Units=O.U


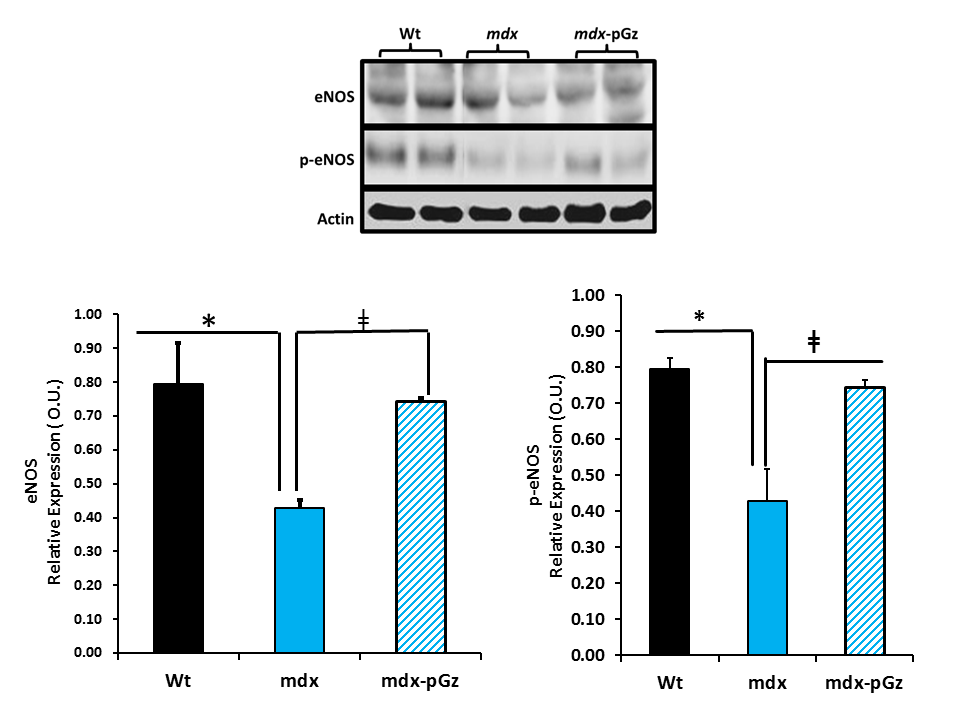


**Protein Expression and Phosphorylation of eNOS Induced by pGz in Mice with Muscular Dystrophy (*md*x)**

The effects of 4 weeks of daily pGz in mice with Muscular Dystrophy on the expression and phosphorylation of eNOS. Muscular Dystrophy mice (*mdx*) had decreased eNOS and p-eNOS compared to control (Wt) (*p< 0.01 Wt vs. mdx). Four weeks of pGz significantly restored both eNOS and p-eNOS levels (ǂ p< 0.001 mdx vs.mdx-pGz). Representative immunoblots of protein expression of eNOS, p-eNOS, and protein loading Actin for Control (Wt), Muscular Dystrophy (mdx), and muscular dystrophy after pGz (mdx-pGz).Optical Units=O.U.

**REFERENCES**

1. Sackner MA, Gummels E, Adams JA. Nitric oxide is released into circulation with whole-body, periodic acceleration. Chest. 2005;127(1):30-9. doi: 10.1378/chest.127.1.30. PubMed PMID: 15653959.

2. Uryash A, Wu H, Bassuk J, Kurlansky P, Sackner MA, Adams JA. Low-amplitude pulses to the circulation through periodic acceleration induces endothelial-dependent vasodilatation. J Appl Physiol (1985). 2009;106(6):1840-7. Epub 2009/03/28. doi: 10.1152/japplphysiol.91612.2008. PubMed PMID: 19325024.

3. Wu H, Jin Y, Arias J, Bassuk J, Uryash A, Kurlansky P, et al. In vivo upregulation of nitric oxide synthases in healthy rats. Nitric Oxide. 2009;21(1):63-8. doi: 10.1016/j.niox.2009.05.004. PubMed PMID: 19481168; PubMed Central PMCID: PMC3135669.

4. Wu H, Uryash A, Bassuk J, Kurlansky P, Giridharan GA, Shakeri M, et al. Mechanisms of Periodic Acceleration Induced Endothelial Nitric Oxide Synthase (eNOS) Expression and Upregulation Using an In Vitro Human Aortic Endothelial Cell Model. Cardiovascular Engineering and Technology. 2012;3(3):292-301. doi: 10.1007/s13239-012-0096-4.

5. Nemzek JA, Xiao HY, Minard AE, Bolgos GL, Remick DG. Humane endpoints in shock research. Shock. 2004;21(1):17-25. doi: 10.1097/01.shk.0000101667.49265.fd. PubMed PMID: 14676679.

6. Toth LA. Defining the Moribund Condition as an Experimental Endpoint for Animal Research. ILAR journal / National Research Council, Institute of Laboratory Animal Resources. 2000;41(2):72-9.

7. Morton DB. A systematic approach for establishing humane endpoints. ILAR journal / National Research Council, Institute of Laboratory Animal Resources. 2000;41(2):80-6. PubMed PMID: 11406701.
